# Industrial Performance of Several *Lachancea thermotolerans* Strains for pH Control in White Wines from Warm Areas

**DOI:** 10.3390/microorganisms8060830

**Published:** 2020-06-01

**Authors:** Cristian Vaquero, Iris Loira, María Antonia Bañuelos, José María Heras, Rafael Cuerda, Antonio Morata

**Affiliations:** 1 enotecUPM. Chemistry and Food Technology Department, ETSIAAB, Universidad Politécnica de Madrid, Avenida Puerta de Hierro 2, 28040 Madrid, Spain; c.vaquero@upm.es (C.V.); iris.loira@upm.es (I.L.); 2 Department Biotecnología-Biología Vegetal, ETSIAAB, Universidad Politécnica de Madrid, Avenida Puerta de Hierro 2, 28040 Madrid, Spain; mantonia.banuelos@upm.es; 3 Lallemand Ibérica, 28521 Madrid, Spain; jmheras@lallemand.com; 4 Comenge Cellars, Curiel de Duero, 47316 Valladolid, Spain; cuerda@comenge.com

**Keywords:** *Lachancea thermotolerans*, acidity, lactic acid, pH control, fresher wines, biotechnology, global warming

## Abstract

In the current scenario of climatic warming, the over-ripening of grapes increases the sugar content, producing flat and alcoholic wines with low acidity, high pH and low freshness. Additionally, a high pH makes wines more chemically and microbiologically unstable, requiring a higher sulphite content for preservation. Some strains of *Lachancea thermotolerans* can naturally lower the pH of wine by producing lactic acid from sugars; this pH reduction can reach 0.5 units. The industrial performance of four selected strains has been compared with that of two commercial strains and with that of *Saccharomyces cerevisiae*. The yeasts were assessed under variable oenological conditions, measuring lactic acid production and fermentative performance at two fermentation temperatures (17 and 27 °C), and in the presence or absence of sulphites (25 and 75 mg/L). Lactic acid production depends on yeast populations, with higher concentrations being reached when the microbial population is close to or above 7-log CFU/mL. A temperature effect on acidification can also be observed, being more intense at higher fermentation temperatures for most strains. Ethanol yield ranged from 7–11% vol., depending on the fermentation conditions (temperature and SO_2_) at day 12 of fermentation, compared with 12% for the *S. cerevisiae* control in micro-fermentations. The production of fermentative esters was higher at 27 °C compared with 17 °C, which favoured the production of higher alcohols. Volatile acidity was moderate under all fermentation conditions with values below 0.4 g/L.

## 1. Introduction

In warm areas, *Vitis vinifera* L. usually produces mature grapes with an excessive sugar content, and therefore, a high potential alcohol together with low acidity and high pH values. This situation is made even worst because of global warming. The pH is a key parameter affecting wine stability and microbiological developments. In turn, the acidity has a strong influence on the sensory quality and freshness of the wine.

To obtain a fresh wine, it is necessary to take into account parameters such as aroma, alcohol content, acidity, colour and more [1,2]. Aromatic freshness is related to the production of acetate esters, produced during fermentation from higher alcohols by the Ehrlich transamination pathway [3], and to some ethyl esters.

There are several ways to increase the acidity of wine, such as the direct addition of tartaric acid [4], the use of ion exchangers which are currently accepted by the OIV (International Organisation of Vine and Wine) [5], but which greatly affect the sensory quality and produce polluting saline effluents, and the fermentative application of some non-*Saccharomyces* yeasts as a biotechnological process to naturally increase the acidity and decrease the pH by the production of organic acids [6]. Certain selected *Saccharomyces* strains can help improve the concentrations of these compounds (weakly), but especially several non-*Saccharomyces* species can increase them during fermentation. Among them, *Lachancea thermotolerans* has proven to be extremely effective [7].

We will focus more on the use of *Lachancea* (*Kluyveromyces*) *thermotolerans* (Lt) [8]; this genus includes, apart from *thermotolerans*, 11 other species: *L. cidri*, *L. dasiensis*, *L. fantastica*, *L. fermentati*, *L. kluyveri*, *L. lanzarotensis*, *L. meyersii*, *L. mirantina*, *L. nothofagi*, *L. quebecensis* and *L. walti*, most of which are ubiquitous [9,10]. *Lachancea thermotolerans* ferments glucose and fructose and also assimilates galactose [11], and is able to use L-lysine as sole source of nitrogen [8]. The use of these species is increasing due to their interesting properties, softening and improving the sensory quality, but especially by lowering the pH by producing lactic acid from sugars [12]. Some strains can produce up to 16 g/L of this acid [13]. These authors reported that acidification can reach 2–3 g/L total acidity and a 0.3 pH reduction when the co-inoculation ratio is 7-log/3-log CFU/mL of *L. thermotolerans*/*S. cerevisiae* [12]. Furthermore, *L. thermotolerans* is a relatively vigorous fermenter, depending on the strain and the physicochemical conditions [14]. As usually happens with non-*Saccharomyces* yeasts, *L. thermotolerans* alone cannot deplete all sugars and therefore requires the sequential or simultaneous addition of another co-starter yeast, generally a *S. cerevisiae* strain [15]. Fermentative acidification by *L. thermotolerans* happens at the beginning of fermentation (days 1 to 4–5) which helps it to be competitive even when there are high wild populations present in the must during fermentation [1,6]. The nitrogen content also influences the lactic acid production; yeast assimilable nitrogen (YAN) values close to 200 mg/L favour lactic acid production [6]. It should be highlighted that the production of lactic acid can come from the sugars in the must, which produces a slight reduction in the alcoholic strength (0.3–0.7% vol.) [1,16]. Additionally, *L. thermotolerans* has been described as a low volatile acidity producer, with a range for most strains of 0.3–0.5 mg/L [1,17,18]. Previous studies have shown the production of aromatic compounds by this yeast, which highlights its role in the production of acetate esters from higher alcohols, fatty acid ethyl esters and enzymatic activities which release terpenes and thiol compounds from cysteine derivatives [3,19,20], and its taste is reminiscent of citrus fruits, with no dairy smell [1]. Recent studies have shown that *L. thermotolerans* can be used to consume acetic acid through the respiration metabolism of yeast [21]. Complementary research has been initiated with this yeast in the area of beer brewing with good results due to the production of fruity esters, with higher glycerol synthesis, and some strains can ferment mannose [22,23]. 

Currently, commercial strains of *L. thermotolerans* are available for oenological applications, e.g., Kt 421 Viniflora^®^ CONCERTO™ (CHR-HANSEN) and LEVEL2 LAKTIA™ (Lallemand, Montreal, Canada). In 2015, our team selected a strain of *Lachancea thermotolerans* (L3.1) in the region of Ribera del Duero (Spain) for its high production of lactic acid and suitable fermentative performance. The aim of this work was to compare the oenological potential of strain L3.1 with other selected *L. thermotolerans* strains from warm areas and with a *Saccharomyces cerevisiae* control strain 7VA.

## 2. Materials and Methods

### 2.1. Yeast Used

The following non-*Saccharomyces* and *Saccharomyces cerevisiae* yeast strains were used in the fermentations: *Lachancea thermotolerans* (Lt) strain L3.1 (Bodegas Comenge, Curiel de Duero, Valladolid, Spain and enotecUPM, Madrid, Spain); strain Kt 421 (CHR-HANSEN, Hoersholm, Denmark, Viniflora^®^ CONCERTO™) (C); strain Laktia (Lallemand, Montreal, Canada, LAKTIA™) (LKT); strains F108 and F111 (isolated at Fontana Cellars, Fuente de Pedro Naharro, Cuenca, Spain); strain A54 (isolated at Altosa Cellars, Tomelloso, Ciudad Real, Spain); *Saccharomyces cerevisiae* strain 7VA (Sc, from enotecUPM, Spain), which was used as the control.

### 2.2. Fermentation Trials

Two batches of fermentations were carried out, one at microscale in 10 mL micro-fermenters and the other at mid-scale in 1 L ISO flasks, to test the performance of the different strains of *Lachancea thermotolerans* through the determination of fermentation kinetics and fermentative power. 

A white juice (*Vitis vinifera* L. cv. Airen) of medium-high pH (pH 3.88; sugars 205 g/L) was used. The juice was obtained by pneumatic pressing and left to settle at a low temperature to clean it and reduce the initial microbial load. Fermentations were performed with volumes of 7 mL of must in micro-fermenters and 1 L in ISO flasks.

Both batches were pasteurised at 100 °C for 10 min (for minimum must degradation). Within the micro-fermentation trial, the must was fermented at two temperatures (17 and 27 °C) and two doses of total SO_2_ (25 and 75 mg/L), all in triplicate. In the 1 L experiment, it was fermented at 17 °C and total SO_2_ at 50 mg/L.

All yeast starters in both trials were grown in YPD (yeast extract, peptone, dextrose agar; 10:20:20 g/L) with two sequential steps of 24 h each to homogenise the populations, and then, 2% *v/v* was inoculated into the must. This inoculation ratio produced a final population of 6-log CFU/mL. The first fermentation trial had all 7 yeasts in triplicate, at two temperatures and with two different amounts of total SO_2_ (mentioned above), and a non-inoculated sample as control. This trial lasted 12 days and the fermentation was monitored by daily weight loss. The second 1 L volume trial was inoculated with the same yeast strains in triplicate and under the conditions already mentioned. This trial lasted 10 days and the basic oenological parameters was monitored daily by FTIR and enzymatic analysis during fermentation.

### 2.3. Yeast Population Counts

Yeast populations were measured by plating in YPD (yeast extract, peptone, dextrose agar; 10:20:20 g/L) on days 3, 6 and 9 during fermentation to compare the viable counts with the production of lactic acid. Yeast counting was performed by spreading 100 µL of serial dilutions of the culture suspensions on YPD agar plates. Colonies were counted after incubation at 27 °C for 3 days.

### 2.4. Oenological Parameters by Infrared Spectroscopy

The equipment OenoFoss™ (FOSS Iberia, Barcelona, Spain) using Fourier-transform infrared spectroscopy (FTIR) was used to identify and quantify major compounds such as residual sugars, organic acids, total acidity (as tartaric acid) and volatile acidity (as acetic acid). This technique also estimates pH value.

### 2.5. pH Determination

The pH of each sample was measured with a Crison micropH 2000 pHmeter (HACH LANGE, Barcelona, Spain). 

### 2.6. Analysis of Lactic Acid

Lactic acid was measured enzymatically using a Y15 enzymatic autoanalyser (Biosystems, Barcelona, Spain).

### 2.7. Colour Parameters Analysed by UV-Visible Spectrophotometry

The absorbance at 420 nm was determined using an Agilent 8453 spectrophotometer (Agilent Technologies S.L., Madrid, Spain) and a 1 mm path length glass cuvette.

### 2.8. Analysis of Fermentative Volatile Compounds Using GC-FID

For this analysis, a gas chromatograph coupled with a flame ionisation detector (GC-FID) was used. Samples were injected after filtration through 0.45 μm cellulose methyl ester membrane filters (Phenomenex, Madrid, Spain). The equipment was an Agilent Technologies 6850 gas chromatograph (Palo Alto, CA, USA). The injection temperature was 250 °C and the detector temperature was 300 °C. The column used was a DB-624 column (60 m × 250 μm × 1.40 μm). The method temperature ramp was 40 °C during the first five minutes, then a linear increase of 10 °C per minute until 250 °C. This temperature was maintained for five minutes. The total runtime of each sample was 40 min. The carrier gas used was hydrogen with a flow rate in column of 2.2 L·min^−1^ and 100 μL of internal standard (4-Methyl-2-pentanol, 500 mg/L) (Fluka Chemie GmbH, Buchs, Switzerland) was added to 1 mL test samples. This method is a variant of the one recommended by the International Organisation of Vine and Wine (OIV) for the analysis of higher alcohols in wine. The limit of detection was 0.1 mg/L. The volatile compounds analysed with this technique were previously calibrated with five-point calibration curves (r^2^): Acetaldehyde (0.999), methanol (0.999), ethanol (0.998), 1-propanol (0.999), diacetyl (0.999), ethyl acetate (0.999), 2-butanol (0.999), isobutanol (0.999), acetic acid (0.958), 1-butanol (0.999), acetoin (0.999), 2-methyl-1-butanol (0.999), 3-methyl-1-butanol/isoamyl alcohol (0.999), isobutyl acetate (0.999), ethyl butyrate (0.999), ethyl lactate (0.999), 2,3-butanediol (0.991), isoamyl acetate (0.999), hexanol (0.999), phenylethyl alcohol (0.994) and phenylethyl acetate (0.999).

### 2.9. Sensory Analysis

A panel of eight experienced tasters (aged between 30 and 60 years, 2 women and 6 men) evaluated the wines bottled and kept under refrigeration for 1 month. The blind tasting took place in the tasting room of the Department of Chemistry and Food Technology of the Polytechnic University of Madrid, equipped with fluorescent lighting and presenting the samples in random order. The wines (30 mL/tasting glass) were served at 12 ± 2 °C in standard odourless tasting glasses. A glass of water was also provided to the panellists for cleaning the palate between samples. Before the generation of a consistent terminology by consensus, three visual attributes, seven for aroma and three for taste, were chosen to describe the wines. Panellists used a scale of 1 to 5 to rate the intensity of each attribute. Low values were “non-perceptible attribute” and, in contrast, high values were “strongly perceptible attribute”. Each panellist also evaluated the overall impression, taking into account olfactory and gustatory aspects, as well as the lack of defects. The tasting sheets also had a final blank space for any additional comments or observations on sensory notes or nuances not previously included as attributes.

### 2.10. Statistical Analysis

Statgraphics v.5 software (Graphics Software Systems, Rockville, MD, USA) was used to calculate means, standard deviation and analysis of variance (ANOVA) and least significant differences (LSD). Significance were set at *p* < 0.05 for the ANOVA matrix F value on the results of the sensory analysis. All treatments were evaluated in triplicate. 

## 3. Results and Discussion

### 3.1. Fermentation Performance

**Figure 1:**
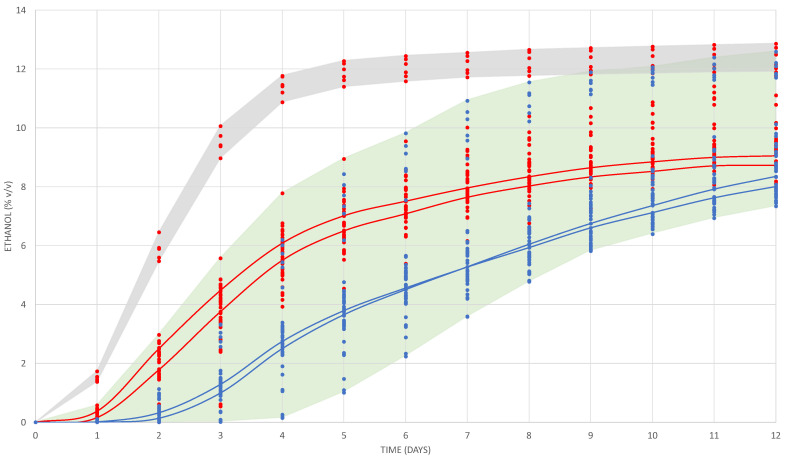
Evolution of the alcoholic content produced during fermentation by the seven yeasts and measured by weigh losses in the micro-fermenters for the four treatments (17 and 27 °C, 25 and 75 mg/L of total SO_2_). The blue dots represent fermentations at 17 °C and red dots at 27 °C. Continuous lines represent the average for *L. thermotolerans* strain L3.1; blue lines at 17 °C (25 and 75 in SO_2_) and red lines 27 °C (25 and 75 in SO_2_). Grey shadow contents the dots for *S. cerevisiae* at 27 °C, and green shadow includes all fermentations with all strains of *L. thermotolerans* plus *S. cerevisiae* at 17 °C.

The fermentation of the 10 mL vials was monitored for 12 days to evaluate the fermentation power and fermentative kinetics. The set of dots shown in Figure 1 describes the evolution of ethanol formation by the different yeast strains.

As expected, the *S. cerevisiae* 7VA (Sc) yeast strain fermenting at high temperature (27 °C) and at both total SO_2_ doses showed the fastest fermentative kinetics (grey shadow, Figure 1). The fermentations of Sc at low temperature (17 °C) and all the fermentations of Lt strains at high temperature started approximately one day later and at a slower rate. Among them, the fermentation rate was variable depending on the strain and the SO_2_ content. Fermentations with all Lt strains at low temperature started 48 h later, when Sc at 27 °C reached 6% *v/v* of ethanol on average. Again, the rate of fermentation among them was variable according to the strain and the SO_2_ content. The red and blue continuous lines represent the fermentative kinetics of Lt strain L3.1 at high and low temperature with both levels of SO_2_.

The highest production of ethanol was reached by the Sc yeast with 12.70 ± 0.19% *v/v* at high temperature and without sulphites (Figure 1). The average ethanol production reached on day 12 by the Lt L3.1 strain (in the four trials) was 8.95 ± 0.64% *v*/*v*, in agreement with previously published values for this species [24,25,26]. It was statistically observed that the yeast with the lowest fermentation power was the F108 strain fermenting in the presence of low SO_2_ and at low temperature (7.74 ± 0.04% *v*/*v*), and the one that showed the highest fermentation power was the CONCERTO yeast (10.69 ± 1.32% *v*/*v*) with low SO_2_ and at high temperature. These results agree with previously reported information where the fermentative performance of the commercial strain Lt CONCERTO is really weak in a range of 9–10% *v/v*, in which it grows with great difficulty [27,28,29]. The Lt L3.1 strain showed an intermediate performance among the pool of strains at high and low temperature.

The fermentations with high temperatures started the stationary phase from day 9–10 for Lt compared with day 6–7 for Sc. The greatest differences in ethanol content over 12 days of fermentation between the strains were observed in the trials with the addition of 75 mg/L of SO_2_. The maximum difference at 27 °C with high SO_2_ was on day 3 with 3.77% *v/v* between the highest and lowest values. The same happened at 17 °C with high SO_2_, in this case on day 5 with 3.42 degrees difference. Using several non-*Saccharomyces* wine yeasts and fermenting at 15 °C, it was observed that Lt was the first to reach an average of 2% *v*/*v* of ethanol at the fourth day of fermentation [30]. This fast start was only improved by *S. cerevisiae*, which reached 2% in 3 days. It has also been observed that the growth of Lt correlates more with Sc at 17 °C than at 27 °C, because Lt shows lower resistance to fermentation temperatures around 30 degrees and, consequently, its growth slows down [18]. The fast start of fermentation by Lt reaching sooner the content of 2% *v/v* of ethanol and the early acidification can be clear parameters to ensure more effective implantation, and with impact on pH reduction when Lt is used in real fermentations in competition with the indigenous yeast population.

### 3.2. Ethanol and Total Acidity at Microscale Fermentation (10 mL) and Middle Scale (1 L)

**Figure 2:**
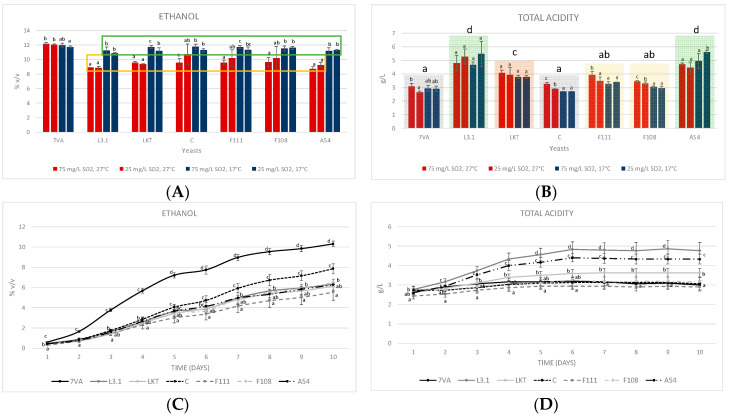
(**A**) Ethanol produced (measured by FTIR) in the 10 mL fermentations at two temperatures (17 °C, blue bars, and 27 °C, red bars) and two SO_2_ contents (25 and 75 mg/L). (**B**) Total acidity in the 10 mL test expressed as tartaric acid. (**C**) Accumulated ethanol formed in the 1 L test (17 °C and 50 mg/L of total SO_2_). (**D**) Total acidity in the 1 L test. Values are means ± sd (*n* = 3). Different letter for the same yeast means significant differences (*p* < 0.05). In section **b**, the averages of each yeast were also statistically compared. The statistic, in section **c** and **d**, reading goes on the same day in the different yeasts.

In the 10 mL micro-fermentations, it was observed that all Lt strains at high temperatures, 27 °C (highlighted with a yellow rectangle, Figure 2A), had the lowest final alcoholic strengths. Regarding the total acidity and according to the statistical analysis of the average values for each yeast, L3.1 and A54 (shadowed in green, Figure 2B) were found to have the highest values due to lactic acid production, F111 and F108 (yellow column) behaved the same, while Laktia (brown column) was slightly higher. Finally, both Sc and CONCERTO (grey column) showed no significant differences, therefore, the production of lactic acid by this Lt strain was marginal. Therefore, temperature is a detrimental parameter for the fermentative performance of LT, even at lower values than reported [18], but conversely, it shows a significant positive effect on acidification for several strains (CONCERTO, F111 and F108, Figure 2B), which are those with lower acidification power. The strains with the highest acidification potential (L3.1, Laktia and A54) were not significantly affected by either temperature or SO_2_ contents.

When the experiment was scaled up to 1 L fermentations (17 °C and 50 mg/L total SO_2_), the yeast kinetics showed a similar performance to that of micro-fermentations. Lt CONCERTO was the fastest growing compared to the F111, which was the slowest (Figure 2C). In terms of acidification, the same pattern was repeated as in the 10 mL test (Figure 2D). Therefore, a 100-fold scale up showed similar parameters in both fermentation yield and acidification performance (Figure 2A,B compared with C,D).

### 3.3. Lactic Acid Production and Microbial Counts

**Figure 3:**
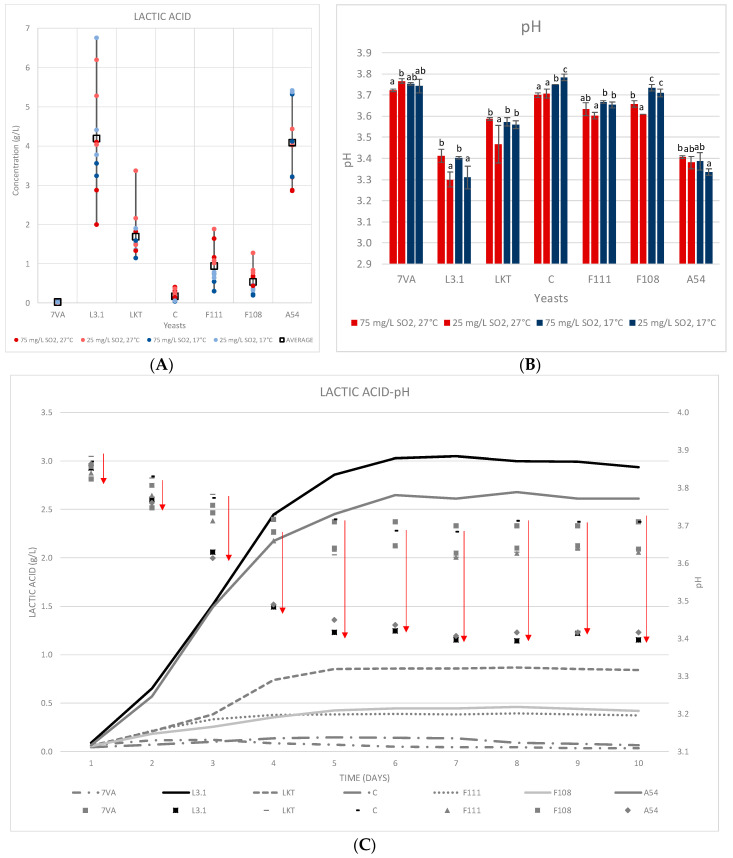
(**A**) The lactic acid produced in the 10 mL fermentations at two temperatures and two SO_2_ contents. (**B**) pH at the end of fermentation. Values are means ± sd (*n* = 3). Different letter for the same yeast means significant differences (*p* < 0.05). (**C**) Accumulated lactic acid formed during fermentation in the 1 L test (17 °C and 50 mg/L of total SO_2_) and the resulting effect on pH.

Lt produces lactic acid from sugars by the expression of its lactate dehydrogenase enzyme. Depending on the amount of lactic acid produced, the final pH of the wine may be modified [31]. Recent studies have shown the great disparity in lactic acid formation (0.26 to 10.54 g/L) by different strains of Lt [32]. At both scales (10 mL and 1 L), the intense acidifying power of the L3.1 and A54 Lt strains was proven, being slightly higher for L3.1 (Figure 3). 

In the 10 mL fermentation, the production of lactic acid was higher (Figure 3A), approximately 1 g/L more than in the 1 L volumes, on average (Figure 3C). Complementary, it is observed that in both fermentations the acidification pattern is always the same; following the order: L3.1 > A54 > Laktia > F111 ≈ F108 > CONCERTO and lastly, the Sc control.

The initial pH was 3.88 since the same must was used in both fermentations and a pH reduction of 0.45 ± 0.4 units was observed in the fermentations with Lt L3.1 and 0.41 ± 0.4 for A54 in the 1 L tests (Figure 3C). The 10 mL test showed an even greater reduction in pH of up to 0.58 ± 0.04 units for Lt L3.1 fermentations and 0.54 ± 0.02 for A54 (Figure 3B). In contrast, the Sc and Lt CONCERTO yeasts only produced 0.12 ± 0.03 and 0.15 ± 0.05 mg/L of lactic acid in the 1 L fermentations, respectively (Figure 3C). The 10 mL test with Sc and CONCERTO also produced the lowest values, with some slight increase in Lt CONCERTO when fermenting at high temperature (27 °C, Figure 3A). It has been observed in recent studies with the same Lt CONCERTO strain that this low production of lactic acid, and therefore, minimal decrease in pH, could increase depending on the starting must [33].

Higher SO_2_ contents (75 mg/L) were found to negatively affect lactic acid production, while the effect of temperature was not significant in general (Figure 3A). In the specific case of the Lt A54 yeast strain, the production of lactic acid was higher at low temperature at both levels of SO_2_. In the 1 L fermentations, the highest content of lactic acid was reached on day 6 and remained stable thereafter (Figure 3C). The effect on pH was subsequent to acidification. Early acidification (days 3 to 5 of fermentation) provides good potential for pH control, even in industrial fermentations, where it is difficult to ensure proper implantation of the Lt inoculum, in agreement with [6]. 

**Figure 4:**
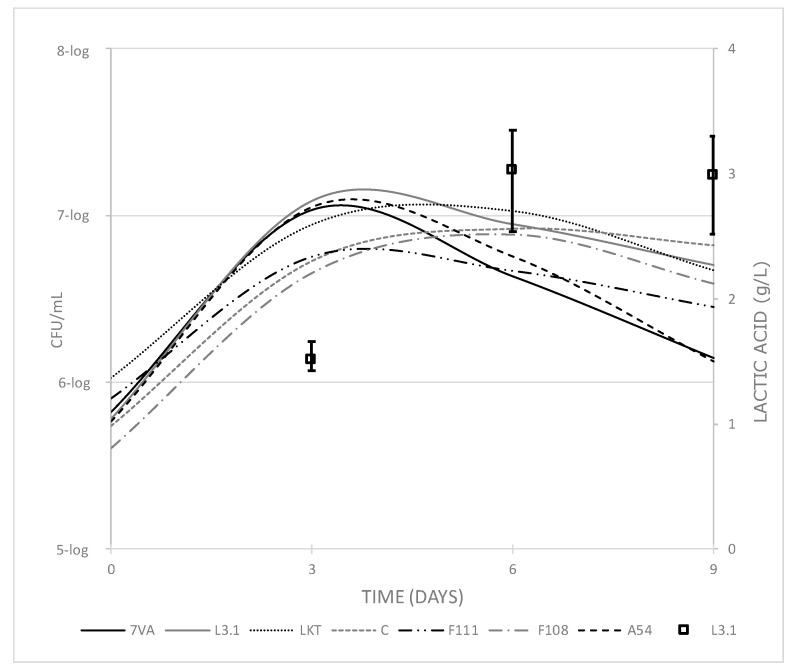
Cell counts of each yeast (CFU/mL) along fermentation compared with the production of lactic acid by Lt L3.1 (squares) and error bars, showing the range of lactic acid values for all the tested strains in the 1 L trial.

As for the yeast populations count, the yeast strains Sc, L3.1, A54 and F111 had their largest population on day 3 in the range 6–7-log CFU/mL, while the other yeast strains had their largest population around day 6, with a population in the same range (Figure 4). There is also a close relationship between yeast counts and lactic acid production. Lt L3.1 was found to have its largest yeast population on day 3, which corresponds closely to lactic acid contents on day 6. Therefore, it seems that a population higher than or around 7-log CFU/mL is needed to start a significant formation of lactic acid and, furthermore, this amount of yeast has been found to be sufficient to contribute to the sensory profile of wines [34], especially in terms of freshness, by lactic acid production.

It is also consistent with a higher production of lactic acid, resulting in a 0.2 pH reduction compared with control, when Lt is inoculated at 7-log CFU/mL [12]; Lt was co-inoculated in a mixed fermentation with Sc at populations lower than 3-log, remaining uncompetitive for 5–7 days with population differences between both species higher than 2-log. Additionally, it has been reported that the proportion and the volume of inoculum (Lt/Sc), whether in mixed or sequential fermentation, should be taken into account for optimal Lt implantation and acidification [12,18]. Therefore, for intense acidification and pH reduction by Lt, the simultaneous 7-log Lt population together with a difference higher than 3-log with competitive Sc can be the optimal situation. There are also published data showing that some Lt strains, even if they cannot finish fermentation, can be viable in the presence of 9% *v/v* ethanol for 10 days [25]. Very recent studies have indicated that using pasteurised musts and sequential fermentation of Lt/Sc, when Sc was added with more than 1% *v/v* of ethanol from the Lt, the lactic acid reached was 10.4 g/L [35], but it should be noted that even in a sequential fermentation, it can leave residual sugars [36]. 

### 3.4. Fermentative Volatiles

Non-*Saccharomyces* yeasts can modify the aromatic profile of wine by producing fermentative volatiles. The acetate esters of higher alcohols and the ethyl esters of medium chain fatty acids are volatile compounds with a strong influence on the positive floral and fruity aroma. In order to evaluate the impact of Lt strains on aroma, we have focussed on the production of fermentative volatiles, which has been grouped into four different categories: higher alcohols, carbonyl compounds, total esters and positive aromatic esters. 

**Figure 5:**
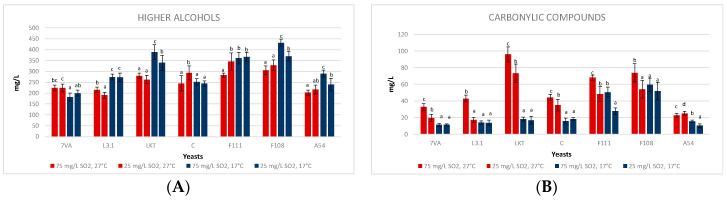

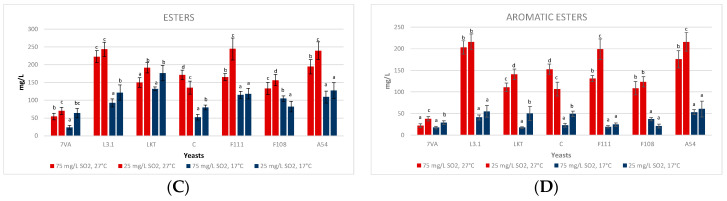
Concentration of (**A**) higher alcohols, (**B**) carbonyl compounds, (**C**) esters and (**D**) aromatic esters in the 10 mL fermentations by the seven strains at two temperatures (17, blue bars, and 27 °C, red bars) and two SO_2_ contents (25 and 75 mg/L). Values are means ± sd (*n* = 3). Different letter for the same yeast means significant differences (*p* < 0.05).

In the 10 mL micro-fermentations, the temperature affected the content of higher alcohols with significant differences in five of the seven strains (Figure 5A). The contents of higher alcohols were a little bit higher at low temperature (17 °C), regardless of the SO_2_ content, except for the fermentations inoculated with Lt CONCERTO and Sc. The effect of SO_2_ content was unclear concerning the formation of higher alcohols. The Lt L3.1 yeast has been previously described as a moderate producer of higher alcohols [1]. The Laktia, F111 and F108 yeasts produced more than 350 mg/L of higher alcohols, which can influence the sensory profile with a more winey and flat aroma [37,38], going through pungent, caramel and fruity smells depending largely on the yeast and the must used [39]. The majority compound was 2-methyl-1-butanol which ranged from 150–200 mg/L in the fermentation of these three strains; however, the flavour threshold of 2-methyl-1-butanol is: 300 mg/L, for isobutanol 75–500 mg/L and for *n*-propanol 300 mg/L [40], higher than the observed concentrations. Similar results were observed in the production of isobutanol (bitter and green aroma [39]), with the mean of the four treatments at 30.62 ± 2.37 mg/L [40]. The 1-butanol is an alcohol that is produced in small quantities, in this case, below 7 mg/L, and high quantities are needed to have aromas of medicine or fruit. The same applies to hexanol, with less than 7 mg/L in cases of fermentations at 27 °C and less than 5 mg/L in cases of 17 °C. Its sensory threshold is above these amounts, with aromas of grass, resin and flowers [41].

Carbonylic compounds (Figure 5B) including acetoin and diacetyl were generally higher at high temperature, especially for Laktia. Its predominant compound was acetoin (86.30 ± 6.67 mg/L), which can confer buttery flavours [42]. No clear differences were seen in these volatiles concerning the SO_2_ effect at low temperatures. However, at high temperatures there is a clear significant trend in the formation of greater amounts of these compounds at high SO_2_ concentration, except for A54 that shows similar concentrations. It was observed that diacetyl, with butter/caramel flavours and a perception threshold between 4 and 12 mg/L (some authors determine that with less quantity it is also detected [20,43]), in the fermentations at 17 °C, the Lt produced less than 5 mg/L, while in the fermentations at 27 °C, higher values with sensory impact were produced [44].

The total esters (Figure 5C), which include ethyl acetate, isobutyl acetate, ethyl butyrate, ethyl lactate, isoamyl acetate and 2-phenylethyl acetate, showed higher concentrations for all Lt strains fermenting at high temperature (27 °C) and with both concentrations of SO_2_. The aromatic esters (Figure 5D) exclude ethyl acetate because it is related to varnish aromas when its concentration is higher than 60 mg/L [18], as it is the case of Laktia (114.44 ± 3.41 mg/L) and F111 (95.10 ± 7.38 mg/L) at high SO_2_ at 17 °C, but also Laktia (126.04 ± 5.78 mg/L) and F111 (93.97 ± 11.08 mg/L) at low SO_2_ and 17 °C. The same does not apply to the average of the other Lt at 27 °C, which is below 30 mg/L of ethyl acetate, while at 17 °C, the average of the Lt is below 55 mg/L [18,40]. There is a clear influence of temperature at low SO_2_ on the formation of esters, except for CONCERTO, which generates more esters in the fermentations at 27 °C and high SO_2_ content (Figure 5C,D). Ethyl butyrate and ethyl lactate appear in greater concentration in Lt fermentations, these esters correspond to pineapple and strawberry/toffee descriptors, respectively [7,41]. Their concentrations in the high-temperature low SO_2_ fermentations were the following: L3.1 (74.30 ± 3.26 mg/L ethyl butyrate and 90.36 ± 9.52 mg/L ethyl lactate), F111 (107.91 ± 14.24 mg/L ethyl butyrate) and A54 (104.79 ± 6.33 mg/L ethyl butyrate and 63.45 ± 8.11 mg/L ethyl lactate). The higher ethyl lactate content is a direct consequence of the strong lactic acid production by most of the Lt strains and agrees with previous works [1] and recent research developed in Lt influence on wine fermentative aroma [35,45]. However, it should be noted that the sensory threshold of ethyl lactate is 150 mg/L which shows sweet, dairy and fruity aromas [1,46], and this value was not reached in the fermentations. It should be added that isoamyl acetate (sweet, banana, fruity aroma with a ripe estry nuance) was not detected and that isobutyl acetate (sweet ethereal banana and tropical fruit aroma) is below 1 mg/L [30,40]. This low production of fruity esters agrees with recent research developed in the influence of Lt on wine fermentative volatiles [33].

**Figure 6:**
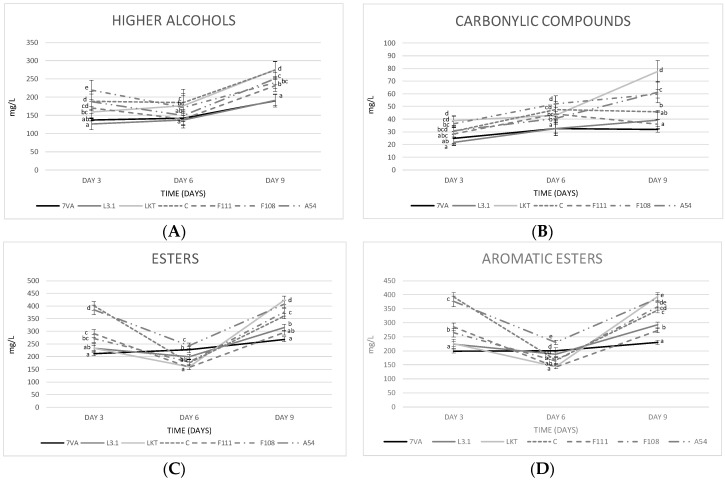
Monitorisation of the fermentative volatiles: (**A**) higher alcohols, (**B**) carbonyl compounds, (**C**) esters and (**D**) aromatic esters on days 3, 6 and 9 of fermentation. Values are means ± sd (*n* = 3). Different letter on the same day of fermentation means significant differences (*p* < 0.05).

In the middle scale trials at 1 L volume, the fermentative volatiles were monitored on days 3, 6 and 9 to see their evolution. It should be noted that the higher alcohols (Figure 6A) were all below 350 mg/L and, therefore, not easily perceived according to their olfactive thresholds. The highest contents were reached in the fermentations by Laktia and CONCERTO with a total of around 275 mg/L, the lowest by Sc and L3.1 with a production of around 190 mg/L, with 2-methyl-1-butanol the majority compound. There was also an intense reduction in isobutanol during fermentation. It was observed that in both 10 mL and 1 L fermentations, 1-propanol was always higher in the average of the Lt fermentations than in the Sc [18]. 

Concerning carbonyl compounds (Figure 6B), yeasts Laktia (77.67 ± 8.5 mg/L) and A54 (59.93 ± 8.46 mg/L) were the most productive at the end of fermentation, while Sc produced the lowest contents (31.98 ± 2.25 mg/L). These compounds generate excessive buttery smells when above 10 mg/L. 

Regarding esters (Figure 6C), the highest amount of ethyl acetate was produced by Laktia (28.35 ± 1.88 mg/L) and F111 (22.77 ± 1.46 mg/L), the first yeast in a similar way to what happened in the 10 mL micro-fermentations. This amount of ethyl acetate was similar to that observed in another recently published article, in which the Lt is also used with an ethyl acetate production of around 20–25 mg/L [47]. In contrast, the Lt CONCERTO has been found to produce less ethyl acetate, around 8 mg/L [48] depending on the fermentation time. In relation to the aromatic esters (Figure 6D), Lt Laktia produced a significant amount of 2-phenylethyl acetate (105.08 ± 7.79 mg/L) with a rose and violet petals aroma descriptor [7,41,49]. The highest ethyl lactate production was seen in the Lt A54 fermentation (35.91 ± 3.20 mg/L), while Sc produced the lowest concentration (21.91 ± 3.14 mg/L).

Ester production by Lt strains was higher compared to Sc. It has also been described that some strains of Lt can release aromatic precursors belonging to the berry, such as thiols and terpenes, thanks to the enzymatic activities β-glucosidase and carbon-sulphur lyase [50,51]. Similar results have been seen in the production of various volatiles (propan-1-ol, butan-1-ol, ethyl acetate) in another study with Lt, but fermenting another type of fruit [52]. Although not included in the figures, it has been found that, as in previous studies, Lt is a low producer of acetaldehyde, generally always below Sc [36]. Acetaldehyde produces aromas due to yeast metabolism such as fruity aromas and notes of nuts or dried fruit [39].

### 3.5. Colour

**Figure 7:**
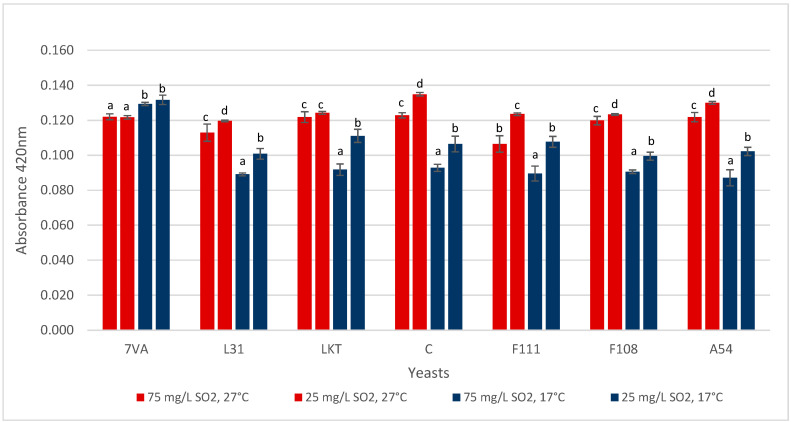
Absorbance at 420 nm (yellow colour) by the wine samples. Values are means ± sd (*n* = 3). Different letter for the same yeast means significant differences (*p* < 0.05).

The pH is a fundamental parameter in the expression of wine colour, and some yeasts, especially Lt, can affect this value [22]. In the 10 mL micro-fermentations, a spectrophotometric analysis performed at 420 nm (Figure 7) to assess this colour parameter, and except for Sc, the other yeasts followed the expected trend in which the higher the fermentation temperature, the higher the colour. It is also likely to be affected by the aeration effect in the small volume, high surface area micro-fermenters. Concerning the SO_2_ content, it was seen, both at high and low temperatures, that a high SO_2_ concentration favours less colour variation or protection against oxidation. 

### 3.6. Sensory Analysis

The wines obtained from the 1 L fermentations were bottled in 0.75 L and sensory evaluated after a month of storage under refrigeration conditions (4 °C). The A54 yeast had the best overall rating with a score of 4.00 ± 0.93 out of 5, and the CONCERTO yeast had the lowest rating with 2.25 ± 0.71 according to the panellists, as it was the weakest in all its parameters. The panellists also found that two yeasts had more acidity, strain L3.1 with 3.75 ± 0.71 and strain A54 with 3.75 ± 1.04, clearly perceived in the tasting. The wine produced by the L3.1 yeast had a great aromatic quality with 3.38 ± 1.19, but it was not statistically significant compared to the other samples, followed by the Lt Laktia with 3.25 ± 1.04, which had a great complexity of aromas [53]. In terms of aromatic intensity, the control Sc obtained the highest score, although again without statistical differences. On the other hand, the Laktia yeast obtained the highest score for the body attribute (3.50 ± 0.76). A comparison with other articles that carried out sensory analysis showed the perfect higher perception of acidity due to Lt, and it was seen that a Lt/Sc co-inoculation generates very interesting sensory parameters [1,18,40]. In agreement with our research, Lt modulates the effect of Sc, producing a more intense freshness in aroma [34] and mouth feel [7]. Globally, the higher ratings were given to the yeasts that produce lactic acid, which increases citric acidity and freshness, in the absence of sugar, and intensifies flavour by improving taste perception [54].

## 4. Conclusions

*Lachancea thermotolerans* is a powerful biotool to reduce the pH naturally in wines, therefore improving the quality and stability of the wine, especially in wines from warm areas. These results also show the inter-strain variability in terms of acidification, but also how relevant oenological parameters such as temperature, SO_2_ content and cell count affect their fermentation performance. The results report useful information to improve the management of wine fermentations with Lt yeasts. According to our results, some Lt strains can strongly reduce wine pH, simultaneously improving the aromatic profile and therefore increasing wine freshness.
